# Supplementary material for: Sweet Immunity: Inulin Boosts Resistance of Lettuce (Lactuca sativa) against Grey Mold (Botrytis cinerea) in an Ethylene-Dependent Manner
Source: Int J Mol Sci. 2019 Feb 28;20(5):1052. doi: 10.3390/ijms20051052 (PMC6429215; doi:10.3390/ijms20051052)

Supplementary figures

Figure S1. The small metabolic sugars Suc, Glc, Fru were tested at 50 mM concentration for possible priming effects, without significant differences.

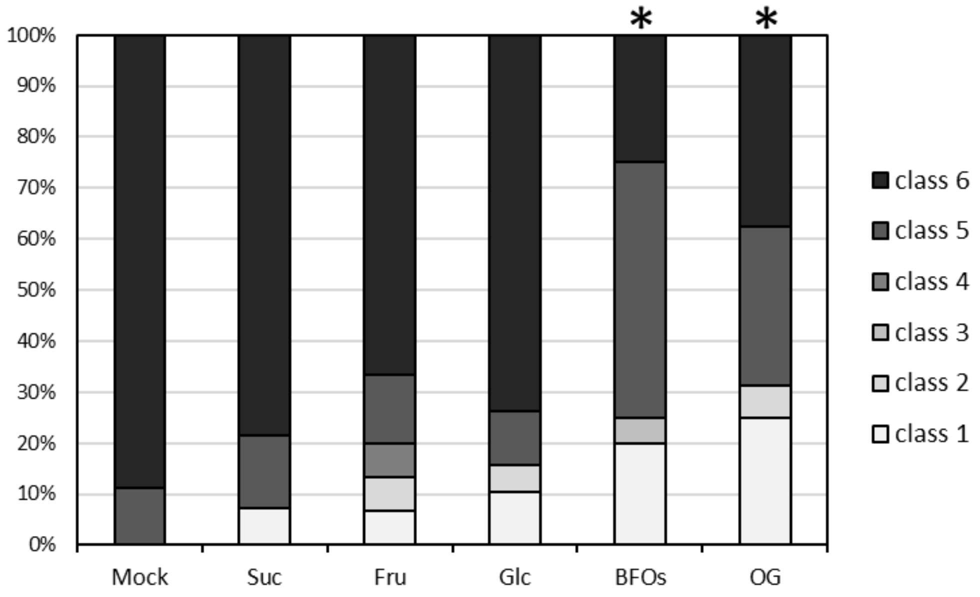

Supplement: Supplementary file 1 [file ijms-20-01052-s001.zip › Figure_S1.pdf]
