# Supplementary material for: Sweet Immunity: Inulin Boosts Resistance of Lettuce (Lactuca sativa) against Grey Mold (Botrytis cinerea) in an Ethylene-Dependent Manner
Source: Int J Mol Sci. 2019 Feb 28;20(5):1052. doi: 10.3390/ijms20051052 (PMC6429215; doi:10.3390/ijms20051052)

Figure S3. Representative pictures of the different treatments for all time points tested for the H_2O_2 accumulation assay.

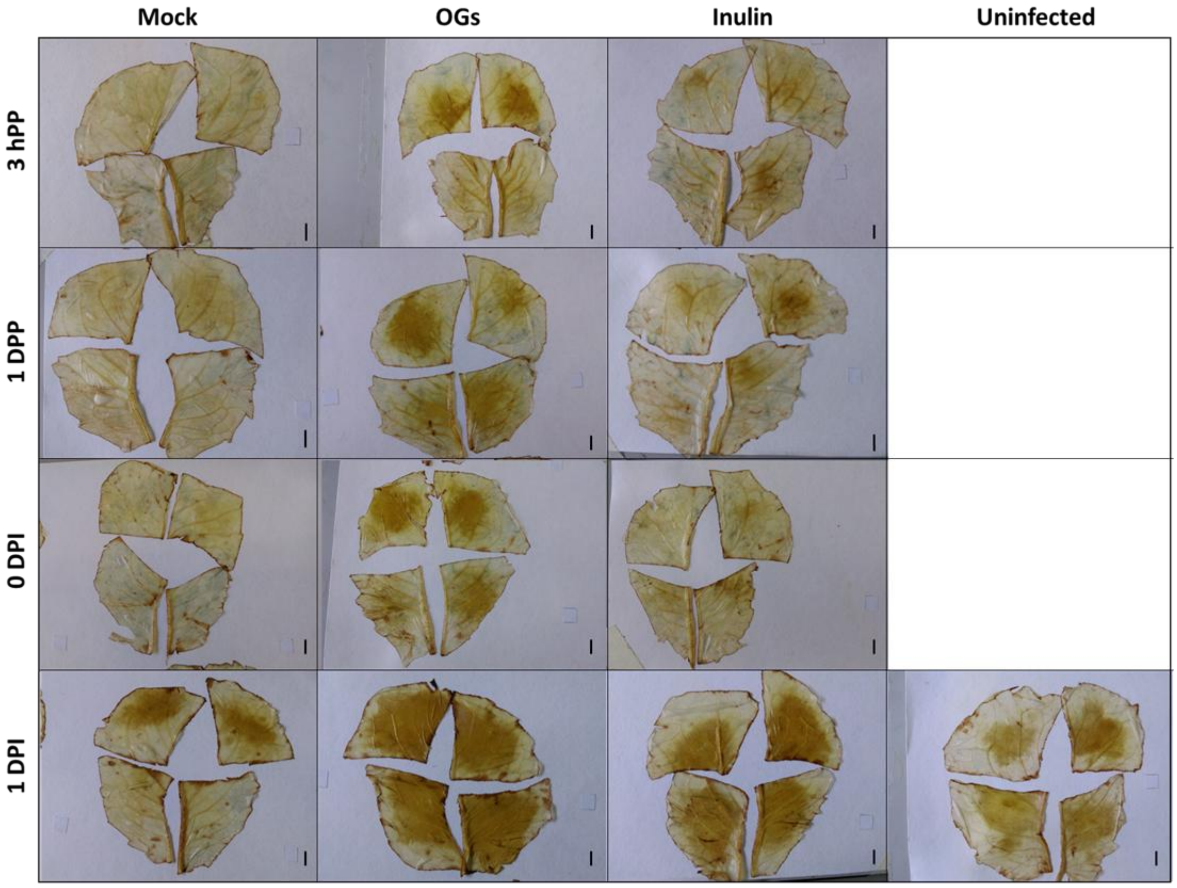

Supplement: Supplementary file 1 [file ijms-20-01052-s001.zip › Figure_S3.pdf]
