# Supplementary material for: Sweet Immunity: Inulin Boosts Resistance of Lettuce (Lactuca sativa) against Grey Mold (Botrytis cinerea) in an Ethylene-Dependent Manner
Source: Int J Mol Sci. 2019 Feb 28;20(5):1052. doi: 10.3390/ijms20051052 (PMC6429215; doi:10.3390/ijms20051052)

Figure S5. Dose-response priming assay with OGs. 0.5 g L^{-1} was determined as the most effective concentration in our pathosystem.

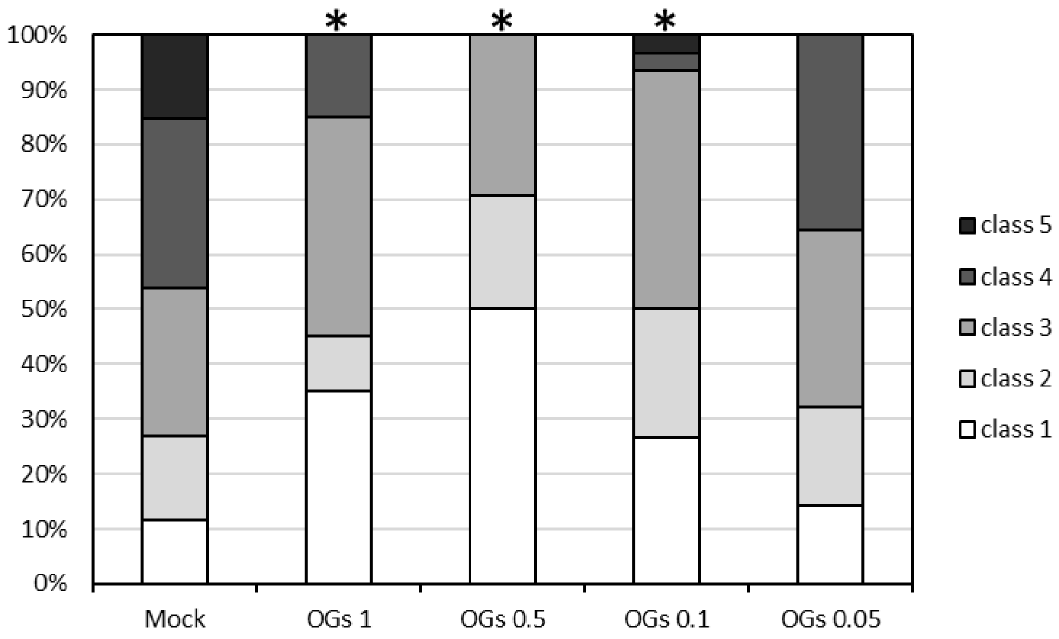

Supplement: Supplementary file 1 [file ijms-20-01052-s001.zip › Figure_S5.pdf]
